# Association Between the Use of Information and Communication Technology Tools and Each Domain of Cognitive Function Among Community-Dwelling Older Adults: A Prospective Cohort Study

**DOI:** 10.7759/cureus.79188

**Published:** 2025-02-17

**Authors:** Kazuki Yokoyama, Hikaru Ihira, Suguru Shimokihara, Yuriko Matsuzaki-Kihara, Atsushi Mizumoto, Hideyuki Tashiro, Hidekazu Saito, Keitaro Makino, Shunpei Katsuura, Kiyotaka Shimada, Kosuke Yama, Ryo Miyajima, Takeshi Sasaki, Nozomu Ikeda

**Affiliations:** 1 Department of Occupational Therapy, School of Health Sciences, Sapporo Medical University, Sapporo, JPN; 2 Department of Physical Therapy, School of Health Sciences, Sapporo Medical University, Sapporo, JPN; 3 Research Fellowship for Young Scientists, Japan Society for the Promotion of Science, Tokyo, JPN; 4 Department of Rehabilitation, Japan Health Care University, Sapporo, JPN; 5 Major of Physical Therapy, Department of Rehabilitation, Faculty of Healthcare and Science, Hokkaido Bunkyo University, Eniwa, JPN; 6 Center for Environmental and Health Sciences, Hokkaido University, Sapporo, JPN; 7 Division of Rehabilitation, Sapporo Medical University Hospital, Sapporo, JPN; 8 Department of Neuropsychiatry, School of Medicine, Sapporo Medical University, Sapporo, JPN; 9 Department of Occupational Therapy, N Field Home-Visit Nursing Station Dune Sapporo, Sapporo, JPN; 10 Psychiatric Rehabilitation Unit, Ebetsu City Hospital, Ebetsu, JPN

**Keywords:** cognitive function, community-dwelling older adults, e-mail, executive function, information and communication technologies, mobile voice calls, video calls

## Abstract

Background: This study aimed to determine the association between the use of information and communication technology (ICT) tools, such as mobile voice calling, e-mail, and video calling, and cognitive function among community-dwelling older adults.

Methods: For the 220 included participants aged ≥65 years, baseline surveys conducted from 2017 to 2018 assessed demographics. Additionally, cognitive function domains were evaluated using the Word List Memory Task, Trail Making Test, and Symbol Digit Substitution Task on a tablet PC. Later, in 2021, follow-up mail surveys assessed the use of ICT tools, including mobile voice calling, e-mail, and video calling.

Results: Multivariate-adjusted logistic regression models revealed that a higher Symbol Digit Substitution Task score was significantly associated with the use of mobile voice calling (odds ratio [OR] = 1.07, 95% confidence interval [CI]: 1.02-1.13), e-mail (OR = 1.09, 95% CI: 1.04-1.15), and video calling (OR = 1.04, 95% CI: 1.003-1.09) after adjusting for covariates.

Conclusions: This study’s findings suggest that processing speed may be related to the use of the three ICT tools assessed in this study among community-dwelling older adults, regardless of tool type. The use of these ICT tools may be challenging for older adults with decreased processing speed. Therefore, preventive interventions should include early recognition of the decline in processing speed and implementation of strategies to compensate for reduced processing speed, such as simplifying processes and habituation procedures, to enable the use of ICT tools by older adults.

## Introduction

The use of information and communication technology (ICT) is increasing worldwide. Various ICT tools such as mobile voice calling, e-mail, and video calling have become widely used, enabling individuals to stay connected even across physical distances. In 2023, internet usage by individuals exceeded 90% in the European Union, Canada, Korea, and other countries [1]. In particular, after the COVID-19 pandemic restricted face-to-face interactions, ICT use rapidly expanded in popularity [2]. In the later stages of life, online activities, including communication, may effectively help maintain social contact and protect mental health [3,4]. ICT use is positively associated with the absence of frailty in individuals aged 75 years and older [5]. Thus, the use of ICT tools is important for reducing social frailty and may contribute to active aging.

In Japan, the penetration rate of mobile devices among older adults aged ≥65 years was 72.7% in 2022, which was greater than that in 2017 [66.6%] [6]. Although the use of ICT by older adults is expected to increase in the future, some older adults may have difficulty using ICT tools. The promotion and use of ICT by older adults to build a digitally inclusive society is under discussion.

Many studies have discussed factors related to ICT use among older adults. In a previous study, the use of messages by e-mail or text was associated with a decreased likelihood of experiencing major depression in older adults but not mild depression [7]. Increased frequency of video calling was also negatively associated with the incidence of depressive symptoms one year later among older adults aged 65-74 years [8]. Some studies have reported that the use of ICT affects the social participation of older adults. The use of e-mail is associated with attending organized activities and volunteering work [9]. Additionally, digital communication technology, especially e-mail and video calling, has consistently been found to improve social support and connectedness and reduce social isolation [10]. Furthermore, ICT, particularly assistive technology, is also used to manage the health status of older adults and contact them during emergencies [11]. Digital health platforms are an encouraging approach to delivering preventive interventions that target cognitive decline and dementia [12,13]. From the above, it can be assumed that a lack of ICT use may lead to poorer psychological well-being and participation restrictions in older adults, as well as a delay in detecting health problems.

Dementia has become a global problem, with the number of patients projected to increase from 57.4 million in 2019 to 152.8 million by 2050 [14]. Recent evidence shows 12 modifiable risk factors in dementia prevention, and those relevant in later life include smoking, depression, and social isolation [15]. Poor cognition in aging has been associated with social isolation and loneliness [16]. Even with cognitive decline, maintaining communication with others is important to prevent social isolation and loneliness in everyday life. Regarding the use of ICTs, cognitive impairment and early stages of dementia have been reported to cause impairment in some instrumental activities of daily living (IADL), including the ability to use the telephone [17,18]. A study assessing executive functions using the clock drawing test and verbal memory showed that they were positively associated with ICT use [19]. Additionally, another study reported that self-reported memory problems interfering with daily activities ≥2 days per week were associated with lower e-mail use [20]. However, the association between different domains of cognitive functioning and the use of specific ICT tools remains unclear.

Clarifying which domains of cognitive function can predict the use of ICT tools can effectively provide support related to cognitive functions when assessing the use of ICT tools. In particular, this study focused on mobile voice calling and e-mail, which are the main methods of communication in Japan [21], as well as video calling, the use of which has expanded rapidly following the COVID-19 pandemic [6,8]. This study aimed to determine the association between the use of ICT tools (mobile voice calling, e-mail, and video calling) and different domains of cognitive function (memory, attention, executive function, and processing speed) among community-dwelling older adults in Japan. The novelty of this study lies in identifying these associations, which may provide insights for developing preventive interventions for cognitive decline and non-use of ICT tools.

## Materials and methods

Participants

**Figure 1:**
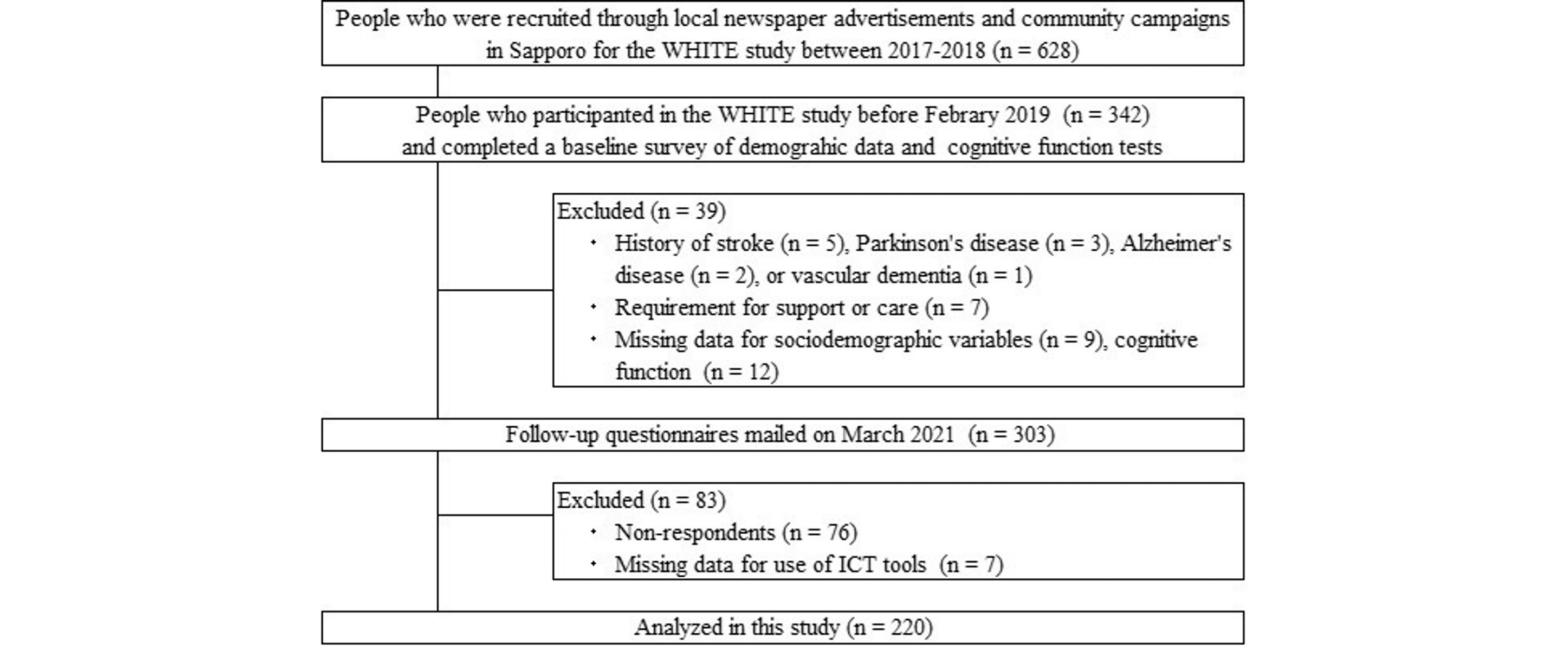
Flowchart of participants inclusion and exclusion ICT: Information and Communication Technology; WHITE: Widely Hokkaido Individual Training for Elderly

Of the 342 participants in the baseline survey of the Widely Hokkaido Individual Training for Elderly (WHITE) study conducted in Sapporo, Hokkaido, in 2017 and 2018, 266 responded to a follow-up questionnaire survey in 2021. A flowchart of the study participants is illustrated in Figure 1. The inclusion criteria for participation at baseline were individuals aged ≥65 years, with no history of cerebral organic disease, no certification of long-term care insurance required, and no difficulties in daily living due to hearing, vision, or speech functions. Participants with a history of stroke (n = 5), Parkinson’s disease (n = 3), Alzheimer's disease (n = 2), or vascular dementia (n = 1) or the need for support or care as certified by the Japanese public long-term care insurance system (n = 7) were excluded. Participants who provided missing or insufficient data on their sociodemographic variables (n = 9), cognitive function scores (n = 12), or use of ICT tools (n = 7) were also excluded.

Data collection

This was a prospective cohort study conducted from September 2017 to August 2021. For the baseline survey, data regarding the basic demographic information were collected, and cognitive function was assessed using a tablet personal computer (PC). In the follow-up survey, approximately three years later, the questionnaire was sent to the participants as a letter. Participants were asked to respond via mail using three ICT tools: mobile voice calling, e-mail, and video calling. 

Use of ICT Tools

Participants were asked the following questions to assess their current use of ICT tools: For mobile voice calls, “Do you currently make voice calls?”; for e-mail, "Do you currently use e-mail?"; and for video calls, "Do you currently make video calls?". The device could be a mobile phone, smartphone, or personal computer. Participants were asked to answer “Yes/No” according to their current situation. For each tool, participants who answered "Yes" were categorized as the "Use group," and those who answered "No" were categorized as the "No use group.” These questions are presented in the Appendix.

Each Domain of Cognitive Function

We used the National Center for Geriatrics and Gerontology-Functional Assessment Tool (NCGG-FAT) to assess cognitive function [22]. An assessor trained in cognitive function measurement assisted the participants in setting up the tablet PC, explained its handling and the cognitive function test, and guided them in understanding the task protocol and recording data. The NCGG-FAT, including its four cognitive function tests (memory, attention, executive function, and processing speed), has been recognized as a reliable and valid tool to assess cognitive function [20] and has been associated with the incidence of dementia [23].

Memory was assessed using the Word List Memory (WLM) task, which included two tablet-based tests: immediate recognition and delayed recall. First, participants were asked to memorize ten words displayed on their tablets for 2 seconds each. They were then presented with 30 words, comprising 10 target words and 20 distractors, and were tasked with identifying the 10 target words. This process was repeated three times. The immediate recognition score was calculated as the mean number of correct selections in the three processes, with scores ranging from 0 to 10. Approximately 20 minutes later, participants were asked to recall the 10 target words and write them on paper. The delayed recall score, ranging from 0-10, was calculated by adding one point for each correctly recalled word within 60 seconds.

Attention was assessed using the tablet version of the Trail Making Test Part A (TMT-A). Participants were required to touch the target numbers shown randomly on the panel as rapidly as possible in consecutive order from 1 to 15. The score was assessed using the completion time for each task in seconds.

Executive function was assessed using the tablet version of TMT-B. The participants were required to alternately touch the target number or letters between consecutive numbers and letters (Japanese Kana) displayed randomly on the screen. The score was assessed using the completion time for each task in seconds.

Processing speed was assessed using the tablet version of the Symbol Digit Substitution Task (SDST). For this task, nine pairs of numbers and symbols were presented at the top of the display. When a target symbol appeared in the center of the display, participants quickly selected the matching number from nine options at the bottom of the display. One point was awarded for each correct selection within the time limit, and the final score was calculated as the total number of correct pairs identified within 90 seconds.

Covariates

The covariates were demographic and lifestyle-related variables that affected the changes in the association between each domain of cognitive function and ICT tools. The questionnaire collected information on these covariates, which included age, sex, living alone status, years of education, and medication status. Living-alone status was identified using a yes or no method. The medication status was recorded as the number of medications taken by the respondents per day.

Data analysis

Participant characteristics were calculated as means and standard deviations (SDs) for continuous variables and numbers and percentages (%) for categorical variables. Each cognitive function test was assessed using means and SDs, and the percentage of each ICT tool used was calculated. Student’s t-tests were conducted to examine the differences in cognitive function test scores at baseline between the ICT tool use and non-use groups. Effect sizes were calculated using Cohen’s d, with 0.2 predicting a small effect size, 0.5 a medium effect size, and 0.8 a large effect size [24]. Three logistic regression models for the use of mobile voice calls, e-mail, and video calls were used to examine the association between the use of the communication tools in the follow-up survey and cognitive function test scores. The independent variables were WLM (immediate recognition and delayed recall), TMT-A, TMT-B, and SDST; the dependent variables were the ICT tools. Age, sex, living alone status, years of education, and medication status were included as covariates in each model. We calculated the odds ratios (ORs) with 95% confidence intervals (95% CIs). Statistical analysis was performed using IBM Corp. Released 2023. IBM SPSS Statistics for Windows, Version 29.0.2.0 Armonk, NY: IBM Corp., and the threshold for statistical significance was set as 0.05.

Ethical considerations

This study was approved by the Sapporo Medical University Ethical Review Board (approval number: 28-2-7; approval date: July 13, 2016). Written informed consent was obtained after the procedures had been fully explained to each participant, and their anonymity was consistently preserved.

## Results

A total of 220 participants were included in the study. Their mean age was 73.7 (SD = 5.6) years at baseline, and 142 (64.5%) were women. Data regarding the participants’ baseline demographics, scores of each component of the NCGG-FAT at baseline, and use of ICT tools during follow-up are summarized in Table 1. Regarding the use of ICT tools, 195 (88.6%) participants used mobile voice calling, 183 (83.2%) used e-mail, and 49 (22.3%) used video calling.

**Table 1:** Characteristics of the participants (n=220) ICT: Information and Communication Technology; WLM: Word List Memory; TMT: Trail Making Test; SDST: Symbol Digit Substitution Task

Variables	Value
Demographic characteristics at baseline	
Age, mean ± SD (years)	73.7 ± 5.6
Female, n (%)	142 (64.5%)
Living alone status, n (%)	53 (24.1%)
Educational history, mean ± SD (years)	13.2 ± 2.3
Medications, mean ± SD (number per day)	2.6 ± 2.7
Cognitive function at baseline	
WLM (Immediate Recognition)	8.1 ± 1.2
WLM (Delayed Recall)	4.5 ± 2.3
TMT-A	20.1 ± 6.3
TMT-B	35.1 ± 22.6
SDST	46.8 ± 9.6
Use of ICT tools at follow-up	
Mobile voice calling, n (%)	195 (88.6%)
E-mail, n (%)	183 (83.2%)
Video calling, n (%)	49 (22.3%)

A comparison of cognitive function tests at baseline using the ICT tools in the follow-up survey is presented in Table 2. For mobile voice calling, the use group had significantly better SDST scores (t (218) = −2.519, p = .018) than the non-use group, indicating a medium effect size (d = −0.674). For e-mail, the use group had significantly shorter TMT-A times (t (218) = 3.040, p = .003) and TMT-B times (t (218) = 2.301, p = .022) and better SDST scores (t (218) = −5.813, p < .001) than the non-use group, indicating a large effect size (d = −1.048). For video calling, the use group had significantly better SDST scores (t (218) = −3.042, p = .003) than the non-use group, indicating a near-medium effect size (d = −0.493).

**Table 2:** Comparison of cognitive function tests at baseline by use of ICT tools at follow-up * p<.05, **p<.01. Student’s t-test. WLM: Word List Memory; TMT: Trail Making Test; SDST: Symbol Digit Substitution Task

Cognitive function tests	ICT tools		t	p-value	Cohen’s d
	No use	Use			
Mobile voice calling	(n = 25)	(n = 195)	-	-	-
WLM (Immediate Recognition)	7.9 ± 1.4	8.1 ± 1.2	−0.868	.386	−.184
WLM (Delayed Recall)	3.7 ± 2.4	4.6 ± 2.3	−1.699	.091	−.361
TMT-A	21.2 ± 4.6	19.9 ± 6.5	0.957	.340	.203
TMT-B	43.2 ± 21.0	34.1 ± 22.7	1.911	.057	.406
SDST	41.2 ± 12.2	47.5 ± 9.0	−2.519	.018*	−.674
E-mail	(n = 37)	(n = 183)	-	-	-
WLM (Immediate Recognition)	7.9 ± 1.4	8.1 ± 1.2	−1.174	.242	−.212
WLM (Delayed Recall)	4.0 ± 2.3	4.6 ± 2.3	−1.401	.163	−.252
TMT-A	22.9 ± 6.3	19.5 ± 6.2	3.040	.003**	.548
TMT-B	42.8 ± 19.4	33.5 ± 23.0	2.301	.022*	.415
SDST	39.0 ± 8.8	48.4 ± 9.0	−5.813	< .001**	−1.048
Video calling	(n = 171)	(n = 49)	-	-	-
WLM (Immediate Recognition)	8.0 ± 1.2	8.3 ± 1.2	−1.504	.134	−.244
WLM (Delayed Recall)	4.3 ± 2.3	4.9 ± 2.4	−1.365	.174	-.221
TMT-A	20.2 ± 6.7	19.5 ± 4.8	.739	.461	.120
TMT-B	35.7 ± 24.6	33.1 ± 13.7	.707	.480	.115
SDST	45.7 ± 9.7	50.4 ± 8.4	−3.042	.003**	−.493

The results of the logistic regression analyses are presented in Table 3. For mobile voice calling, the use group showed significant associations with the SDST score (OR = 1.07; 95% CI: 1.02-1.13, p = .012) compared with participants in the non-use group after adjusting for age, sex, living alone status, years of education, and medication status. For e-mail, the use group showed significant associations with the SDST score (OR = 1.09; 95% CI: 1.04-1.15, p < .001) after adjusting for age, sex, living alone status, years of education, and medication status. For video calling, the use group showed significant associations with the SDST score (OR = 1.04; 95% CI: 1.003-1.09, p = .048) after adjusting for age, sex, living alone status, years of education, and medication status.

**Table 3:** Association between use of ICT tools and cognitive function tests among community-dwelling older adults * p<.05, **p<.01. ICT: Information and Communication Technology; WLM: Word List Memory; TMT: Trail Making Test; SDST: Symbol Digit Substitution Task Adjusted for age, sex, living alone status, year of education, and medication status (at baseline).

Cognitive function tests	ICT tools		
	Mobile voice calling	E-mail	Video calling
WLM (Immediate Recognition)	1.06 (0.74–1.53)	1.03 (0.74–1.45)	1.10 (0.81–1.50)
WLM (Delayed Recall)	1.10 (0.90–1.34)	0.99 (0.82–1.19)	1.01 (0.87–1.18)
TMT-A	0.98 (0.92–1.05)	0.95 (0.90–1.00)	1.00 (0.94–1.05)
TMT-B	0.99 (0.97–1.004)	0.99 (0.98–1.01)	1.00 (0.98–1.02)
SDST	1.07 (1.02–1.13)*	1.09 (1.04–1.15)**	1.04 (1.003–1.09)*

## Discussion

The findings of this prospective cohort study suggested that SDST is associated with the use of mobile voice calling, e-mail, and video calling. To our knowledge, this is the first study to show processing speed is associated with ICT use in community-dwelling older adults. The participants in this study were adults aged 65 years or older living in Hokkaido, and their average age at follow-up was over 75 years. In a previous study conducted during the same period, mobile device adoption was reported in 81.7% of those aged ≥65 years and 72.9% of those aged ≥75 years [21]. Similarly, e-mail adoption was reported in approximately 66.3% of those aged ≥65 years and 55.5% of those aged ≥75 years [21]. The mobile phone usage rate in this study was over 90%, and the e-mail usage rate was over 80%, suggesting that the participants in this study had higher usage rates than the national average.

Among the various types of cognitive function tests, processing speed scores as measured by the SDST were shown to be associated with the use of mobile voice calling, e-mail, and video calling in follow-up surveys among community-dwelling older adults, regardless of the differences in the type of ICT tools. This may be related to the difficulty associated with the cognitive test and the characteristics of the domains. In previous studies, processing speed measured by the SDST was reported to be associated with unsafe driving acts during an on-road test [25] and motor-cognitive dual-task test [26]. However, no association was found between the cognitive function tests in the other domains. In addition, the SDST had the highest predictive value for the incidence of dementia compared to other cognitive function tests such as word list memory, TMT-A, and TMT-B, suggesting that processing speed may be a helpful factor for assessing dementia in community settings [23]. Thus, it can be assumed that the SDST, which assesses processing speeds, is associated with more complex daily functioning than verbal memory, TMT-A, and TMT-B. As the use of ICT tools involves complex processes, such as operating the device and communicating with others, the association was found only for SDST, which may be useful for the early detection of dementia risk in the community [23].

In summary, among the cognitive functions of older adults, only processing speed was found to be associated with the use of ICT tools for voice calling, e-mailing, and video calling after three years. Early detection of reduced processing speed may be important for continued ICT use among older people. Assessment of IADLs due to cognitive decline has shown that focusing on the processes that are impaired can guide interventions [18]. Therefore, identifying processes that are slowing down may be important for the continued use of ICT tools. In addition, as procedural learning appears to remain intact in mild cognitive impairment and Alzheimer kind of dementia [27], it is important to fix and habituate simplified procedures early on in the process of ICT use to enable interventions using procedural memory.

However, the results of this study revealed that memory, attention, and executive functions did not predict the use of ICT tools in the follow-up surveys after adjusting for covariates. A previous study suggested that the levels of episodic memory predicted online-based applications and devices to obtain information and improve communication two years later, but not the levels of executive functioning [28]. However, that study evaluated the overall use of ICT tools, including content other than phone calls, e-mail, and video calls, and did not analyze individual ICT tools separately. Therefore, it can be inferred that the results of the study differed from those of previous studies. In addition, the participants in the present study showed immediate and delayed reaffirmation and delayed replay results similar to or better than the reference values reported in previous studies [22,29]. It is assumed that this was because many participants in this study had good levels of memory, attention, and executive function, which did not predict their use of ICT tools. The results also suggest that the use of ICT tools can be implemented even when memory and attention functions are slightly reduced.

This study had several limitations. First, the participants had applied themselves to participate in this study conducted in Hokkaido, Japan, and the number of participants was small. As it is possible that different results could be obtained if the survey was conducted in other regions, a future randomized large-scale survey that considers regional characteristics is desirable. Second, the ICT tools were limited to three types, and their use was self-reported. This study did not examine the devices used by the participants, their frequency of use, how they used the devices, whether they needed help in using them, or with whom they communicated. Moreover, the association with the impact of another tool was also not evaluated. In the future, it will be necessary to clarify the relationship between these factors and cognitive function. Third, although it was possible to predict that low SDST scores would be associated with the underutilization of ICT tools, the extent of underutilization of ICT tools and their association with the incidence of disease remain unclear. Cognitive decline has negative psychological effects, such as depressive symptoms [30] and apathy [31], in older adults. A definition of cognitive communication disorders has been proposed, and it is well-known that cognitive decline significantly reduces communication and limits social participation [32]. In the future, clarifying the association between ICT use and cognitive function, including psychological issues not focused on in this study, is essential.

## Conclusions

This study’s findings suggest that processing speed as measured by the SDST is associated with the use of mobile voice calling, e-mail, and video calling among community-dwelling older adults. Such ICT tools may be difficult to use in older adults with reduced processing speeds. Assistance that examines ways to compensate for reduced processing speeds, such as simplifying processes and habituation procedures, may be required for the use of ICT tools by older adults. In the future, we aim to explore the behavioral factors related to the use of ICT tools, as they can lead to both frailty prevention and improved well-being. The above findings highlight the need for future research to explore interventions to improve processing speed and ICT use among older people and identify the interrelationship between them.

## Appendices

Questionnaire 

**Table 4:** Questionnaires for the ICT tools ICT: Information and Communication Technology

Please answer “Yes” or “No” to whether you currently use the following ICT tools on the devices you own (mobile phone, smartphone, personal computer, etc.).	
Questionnaire	Response items
Do you currently make voice calls?	Yes or No
Do you currently use e-mail?	Yes or No
Do you currently make video calls?	Yes or No
